# Impact of anticoagulation therapy on the cognitive decline and dementia in patients with non‐valvular atrial fibrillation (cognitive decline and dementia in patients with non‐valvular atrial fibrillation [CAF] trial)

**DOI:** 10.1002/joa3.12781

**Published:** 2022-09-19

**Authors:** Thomas Jared Bunch, Heidi May, Michael Cutler, Scott C. Woller, Victoria Jacobs, Scott M. Stevens, John Carlquist, Kirk U. Knowlton, Joseph B. Muhlestein, Benjamin A. Steinberg, Jeffrey L. Anderson

**Affiliations:** ^1^ Intermountain Heart Institute and Department of Medicine, Intermountain Medical Center Murray Utah USA; ^2^ Department of Internal Medicine, Division of Cardiology University of Utah School of Medicine Salt Lake City Utah USA; ^3^ Department of Internal Medicine University of Utah School of Medicine Salt Lake City Utah USA

**Keywords:** anticoagulants, atrial fibrillation, cognition, dementia, stroke

## Abstract

**Background:**

Atrial fibrillation (AF) is associated with a risk for cognitive impairment and dementia, which is more pronounced in patients with a history of clinical stroke. Anticoagulation use and efficacy impact long‐term risk of dementia in AF patients in observational trials.

**Methods:**

The cognitive decline and dementia in patients with non‐valvular atrial fibrillation (CAF) Trial was a randomized, prospective, open‐label vanguard clinical study with blinded endpoint assessment involving patients with moderate‐ to high‐risk (CHADS2 or CHA2DS2‐Vasc scores of ≥2) non‐valvular AF assigned to dabigatran etexilate or warfarin. The primary endpoint was incident dementia or moderate cognitive decline at 24 months.

**Results:**

A total of 101 patients were enrolled [mean age:73.7 ± 6.0 years, male: 54(53.5%)]. Prior stroke and stroke risk factors were similar between groups. Average INR over the study was 2.41 ± 0.68 in the warfarin group. No patient experienced a stroke or developed dementia. Mini‐Mental Status Evaluation, Hachinski Ischemic scale, cognitive subscale of the Alzheimer's Disease Assessment Scale, Disability Assessment for Dementia, Quality of Life Improvement as assessed by Minnesota Living with Heart Failure Scale and the Anti‐Clot Treatment Scale Quality of Life Survey scores did not vary at baseline or change over 2 years. Biomarker analysis indicated a similar efficacy of anticoagulation strategies.

**Conclusion:**

Use of dabigatran and well‐managed warfarin therapy were associated with similar risks of stroke, cognitive decline, and dementia at 2 years, suggestive that either strategy is acceptable. The results of this Vanguard study did not support the pursuit of a larger formally powered study.

## Graphical abstract

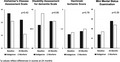

## 1 BACKGROUND

Atrial fibrillation (AF) is associated with a risk for stroke, transient ischemic attack, and more recently with other forms of brain injury such as cognitive impairment and dementia. In observational studies, the relative risk of cognitive impairment and dementia among patients with AF and a prior stroke is estimated between 2.43–2.70.^1^, ^2^

The Swiss Atrial Fibrillation (Swiss‐AF) cohort evaluated for the presence of stroke (clinical and subclinical) in a baseline cohort of enrollees with AF that underwent MRI imaging (*n* = 1737 patients). Large cortical and noncortical infarcts were found in 22% of patients, small noncortical infarcts were found in 21%, microbleeds in 22%, and white matter lesions in 99%.^3^ These infarcts, whether labelled as clinical or subclinical were associated with cognitive dysfunction. A more recent analysis from the Swiss‐AF cohort reported the outcomes of 1227 patients that had a baseline brain MRI and another 2 years later. In this analysis, 2.3% patients had a new clinical stroke or TIA. However, at least one infarct was detected in 5.5% patients on the follow‐up MRI.^4^ The presence of a new stroke/cerebral infarct correlated with cognitive dysfunction, whether the event was deemed clinical or subclinical by symptomatic diagnosis. These studies derived from contemporarily managed populations with AF continue to highlight the critical pathway of cerebral ischemic events as a contributing mechanism for cognitive decline and dementia in patients with AF.

Although there are randomized control data that show direct oral anticoagulants (DOAC) lower risk of stroke and intracranial bleeding compared to warfarin therapy,^5^, ^6^, ^7^ there remains a lack of data to determine if DOAC therapy may lower risk of cognitive decline and dementia in comparison to warfarin therapy. There are mixed data in observational studies that have assessed if DOACs, that are more predictable and effective in prevention of stroke and intracranial bleeds, may further lower risk compared to warfarin.^8^, ^9^

Dabigatran etexilate, an oral direct thrombin inhibitor, was studied versus warfarin in patients with non‐valvular AF.^6^ Over a median follow‐up of 2 years, dabigatran at 150 mg BID compared to warfarin lowered the risk of stroke of systemic embolism and reduced risk of hemorrhagic stroke. We also found in a community analysis of DOAC therapies, dabigatran etexilate had the lowest observed dementia rates despite the longest duration of follow‐up.^9^

A recent consensus statement called for more prospective data regarding the use, adherence, and efficacy of anticoagulation in AF to prevent cognitive decline and dementia.^10^ The Cognitive Decline and Dementia in Atrial Fibrillation Patients (CAF) Trial (ClinicalTrials.gov Identifier: NCT03061006) was proposed to determine if AF patients randomized to dabigatran etexilate will have long‐term higher cognition scores and lower rates of dementia compared to dose‐adjusted warfarin (INR: 2.0–3.0).

## 2 METHODS

### 2.1 Study objectives and design

This was a randomized, prospective, open‐label clinical study with blinded endpoint assessment. The methodology and design have been published elsewhere.^11^ Patients (>65 years) with moderate‐ to high‐risk (defined as a CHADS2 score or CHA2DS2‐Vasc score of ≥2)^12^ non‐valvular AF were randomly assigned to standard dosing dabigatran etexilate or warfarin, adjusted to a target INR of 2.0–3.0 were enrolled from March 30, 2017 to March 25, 2019 for their initial anticoagulation strategy as part of their routine management of AF.

The primary functional endpoint of this study was to demonstrate whether long‐term anticoagulation therapy with dabigatran etexilate (150 mg BID or 75 mg BID, dose based upon renal clearance) would reduce incident dementia and worsening cognitive decline compared to dose‐adjusted warfarin. Incident dementia was defined as a formal diagnosis of dementia by a neurologist. Subjects that scored <24 upon completing the mini‐mental status evaluation (MMSE) and reported memory loss affecting quality of life were referred to neurology for further evaluation of dementia. Cognitive decline was determined by measuring the change from baseline to study conclusion on the 11‐item cognitive subscale of the Alzheimer's Disease Assessment Scale (ADAS, with scores ranging from 0 to 70 and higher scores indicating greater impairment) and the Disability Assessment for Dementia (DAD, with scores ranging from 0 to 100 and higher scores indicating less impairment). An increase in ADAS‐cog11 of >30% was considered significant for moderate cognitive decline. In subjects that scored <50% on the DAD, there was a direct correlation with global deterioration scales and scores. Subjects with a 30% decrease in DAD score or those with a score < 50% were considered to have moderate cognitive decline. Secondary endpoints included new stroke (clinical or subclinical) or transient ischemic attack (TIA) and intracranial bleed.

We previously published the power analysis based upon background community work to detect a difference in the primary endpoint of 1100 study participants.^13^ In regard to the enrollment need and uncertainty regarding the true impact of different anticoagulants on cognitive performance in patients with AF, we first chose to enroll 120 subjects using a vanguard study design. This number of subjects was chosen to provide a more precise understanding of the incidence of both dementia and moderate cognitive decline in each anticoagulation group. The data derived from the vanguard analysis informed the primary endpoint analysis and optimization of study sample size as well as decision making regarding enrollment, retention, and study feasibility.

The study was a single center trial that was conducted at Intermountain Medical Center in Murray, Utah approved by the Intermountain Heart Scientific Review Board and the Internal Review Board. A data safety monitoring board was created and reviewed patient events and study progress at predetermined intervals.

### 2.2 Subject selection criteria

All patients had to be able to take oral anticoagulation, complete serial testing of cognition and functional status, and have a moderate risk of stroke at enrollment (CHADS2 score or CHA2DS2‐Vasc score of ≥2). Patients were excluded if they did not meet inclusion criteria, had severe renal dysfunction (CrCl <15 mL/min) that impaired use of dabigatran anticoagulation, or had a diagnosis of dementia.

### 2.3 Time and events schedule

Subject medical records were collected as part of usual medical care prior to enrollment to obtain baseline demographics for the 6 months preceding randomization. The six cognitive and clinical assessment questionnaires were administered at the baseline visit, and repeated at the 6‐, 12‐, 18‐ and 24‐month visits were the Mini‐Mental Status Evaluation (MMSE), Hachinski Ischemic scale (HIS), cognitive subscale of the Alzheimer's Disease Assessment Scale (ADAS), Disability Assessment for Dementia (DAD), Quality of Life Improvement as assessed by Minnesota Living with Heart Failure Scale and the Anti‐Clot Treatment Scale Quality of Life Survey. All testing was performed by experienced research coordinators that received institutional assessment and certification regarding education, efficacy and consistency before they begin to administer the tools.

In each group, 10 subjects were selected to undergo a cranial MRI at baseline to determine brain volume and characteristic changes representative of microbleeding, with a repeat MRI at 24 months. These subjects were selected at time of enrollment until all slots have been filled (i.e., first 10 subjects in each treatment arm who are willing and able to undergo the procedure). Brain volume was defined as per routine description.^14^, ^15^, ^16^

#### 2.3.1 Additional serum tests at baseline

Biomarker testing associated with stroke, intracranial bleed, thrombosis and dementia performed at enrollment and the 24‐month visit included brain natriuretic peptide (BNP), troponin, growth differentiation factor (GDF)‐15, cystatin‐C, D‐dimer, matrix metalloprotinease (MMP)‐9, chemokine ligand 23 (CCL23), endothelial cell adhesion molecule (ESAM), plasma von Willebrand factor, C reactive protein (CRP), prothrombin fragment 1 + 2, P‐selectin, factor VIII, protein C, protein S, anti‐thrombin III, anti‐beta‐2 glycoprotein‐1 IgG and IgM, anticardiolipin IgG and IgM, and lupus anticoagulant panel. Because the most common gene polymorphism associated with sporadic microbleeds is the Apolipoprotein E (APOE) gene on chromosome 19, to control for this variable we will test for this gene as well as and the APOE ɛ2 and ɛ4 alleles which have each been independently associated with lobar microbleeds.^17^, ^18^, ^19^ Neprilysin and the single‐nucleotide polymorphism rs6656401 within the Complement Receptor‐1gene that are associated with severe cerebral amyloid angiopathy will also be assessed.^20^, ^21^

### 2.4 Statistical analysis

Descriptive statistics were used to report the recruitment, study subject retention, budget adherence and likelihood of obtaining the desired outcomes from the pre‐specified power analysis, safety, and identification of adverse effects.

For the primary outcomes, 2‐year incident dementia and whether there was a significant change in ADAS‐cog11 (increase of >30%) and DAD (score < 50% and/or a 30% decrease) scores were evaluated using the chi‐square statistic. In addition, change scores for ADAS‐cog11 and DAD (2‐year survey score minus baseline survey score) utilized the Student's *t*‐test and the Mann Whitney Rank Sum test. Comparisons of change scores between baseline and other time points (i.e., 6, 12, and 18 months) was used the Student's *t*‐test and Mann Whitney Rank Sum test to determine significant differences at these time points. Analyses was performed on an intention‐to‐treat basis.

## 3 RESULTS

The baseline demographics of the study population are shown in Table 1. Among the 101 patients randomized 50 were randomized to dabigatran (49.5%) and 51 to warfarin (50.5%). The groups were balanced with the exception of coronary artery disease and treatment of coronary artery disease that was more prevalent in the warfarin treated group.

**TABLE 1:** Baseline characteristics of all randomized patients stratified by treatment arm

	Dabigatran	Warfarin	*p*‐value
Age (years)	73.4 ± 5.5	74.0 ± 6.5	.60
Sex (male)	26 (52.0%)	28 (54.9%)	.84
Race (white)	50 (100%)	50 (98.0%)	1.00
Ethnicity (non‐Hispanic/Latino)	50 (100%)	51 (100%)	1.00
Hypertension	31 (62.0%)	35 (68.6%)	.48
Diabetes	16 (32.0%)	22 (44.0%)	.30
Depression	16 (32.0%)	12 (23.5%)	.34
Heart failure	11 (22.0%)	8 (16.0%)	.44
Renal disease	8 (16.0%)	14 (28.0%)	.15
Atherosclerosis	6 (12.0%)	10 (19.6%)	.30
CAD	8 (16.0%)	18 (36.0%)	.02
Prior MI	3 (6.0%)	9 (18.0%)	.07
Prior revascularization	1 (2.0%)	8 (16.0%)	.03
Prior stroke	6 (12.0%)	6 (11.8%)	.97
Prior TIA	4 (8.0%)	4 (8.0%)	1.00
History of vascular disease	3 (6.0%)	5 (10.0%)	.72
AF type, *n* = 67			.28
Paroxysmal	27 (81.8%)	32 (94.1%)	
Persistent	4 (12.1%)	1 (2.9%)	
Long‐standing persistent	2 (6.1%)	1 (2.9%)	
Prior ablation	5 (10.0%)	4 (8.0%)	1.00
Pacemaker	6 (12.0%)	7 (14.0%)	.77
Defibrillator	2 (4.0%)	2 (4.0%)	1.00
History of cancer	6 (12.0%)	13 (26.0%)	.07
Sleep apnea	16 (32.0%)	17 (34.0%)	.83
BMI (kg/m^2^)	30.7 ± 9.8	29.3 ± 7.8	.44
Systolic blood pressure	131.5 ± 16.3	131.6 ± 22.5	0.98
Diastolic blood pressure	78.7 ± 12.2	76.1 ± 16.9	.38
CHA_2_DS_2_‐VASc categories, *n* = 100			.38
≤1	3 (6.0%)	2 (4.0%)	
2–4	37 (74.0%)	32 (64.0%)	
≥5	10 (20.0%)	16 (32.0%)	
ATRIA bleeding risk categories, *n* = 74			.29
≤1	19 (51.4%)	15 (40.5%)	
2–4	15 (40.5%)	21 (56.8%)	
≥5	3 (8.1%)	1 (2.7%)	
Dabigatran starting dose			
75 mg BID	3	—	—
150 mg BID	44	—	—
Withdrew before start	3	—	—

Abbreviations: AF, atrial fibrillation; BMI, Body mass index; CAD, coronary artery disease; MI, myocardial infarction; TIA, transient ischemic attack.

Of the patients enrolled in the study, 64% of those randomized to dabigatran and 61% of those randomized to warfarin completed the study. Supplemental Table S1 shows the reasons provided for discontinuing drug therapy.

Table 2 shows the baseline demographics of the study population that completed the study. In the dabigatran group, more patients had worse subtypes of AF (persistent and longstanding persistent) compared to the group treated with warfarin and a history of cancer was more common in the warfarin group.

**TABLE 2:** Baseline characteristics among patients who completed the study stratified by treatment arm

	Dabigatran, *n* = 32	Warfarin, *n* = 31	*p*‐value
Age (years)	72.8 ± 5.7	73.5 ± 6.3	.64
Sex (male)	17 (53.1%)	19 (61.3%)	.51
Race (white)	32 (100%)	31 (100%)	—
Ethnicity (non‐Hispanic/Latino)	32 (100%)	31 (100%)	—
Hypertension	22 (68.8%)	20 (64.5%)	.72
Diabetes	9 (28.1%)	12 (38.7%)	.37
Depression	13 (40.6%)	7 (22.6%)	.12
Heart failure	6 (18.8%)	2 (6.5%)	.14
Renal disease	4 (12.5%)	9 (29.0%)	.11
Atherosclerosis	5 (15.6%)	7 (22.6%)	.48
CAD	6 (18.8%)	11 (35.5%)	.14
Prior MI	3 (9.4%)	4 (12.9%)	.71
Prior revascularization	1 (3.1%)	6 (19.4%)	.05
Prior stroke	3 (9.4%)	5 (16.1%)	.47
Prior TIA	3 (9.4%)	2 (6.5%)	1.00
History of vascular disease	3 (9.4%)	1 (3.2%)	.61
AF type, *n* = 42			.04
Paroxysmal	15 (75.0%)	22 (100%)	
Persistent	4 (20.0%)	0 (0%)	
Long‐standing persistent	1 (5.0%)	0 (0%)	
Prior ablation	2 (6.3%)	4 (12.9%)	.43
Pacemaker	6 (18.8%)	4 (12.9%)	.73
Defibrillator	2 (6.3%)	1 (3.2%)	1.00
History of cancer	2 (6.3%)	8 (25.8%)	.04
Sleep apnea	11 (34.4%)	12 (38.7%)	.72
BMI (kg/m^2^)	33.3 ± 9.0	29.9 ± 9.5	.16
Systolic blood pressure	130.9 ± 14.7	131.6 ± 20.8	.87
Diastolic blood pressure	78.3 ± 11.8	75.2 ± 10.8	.27

Abbreviations: AF, atrial fibrillation; BMI, Body mass index; CAD, coronary artery disease; MI, myocardial infarction; TIA, transient ischemic attack.

The 2 year results data and secondary endpoints of Stroke or Transient ischemic attack (TIA), intracranial bleed, and changes from baseline scores on the mini‐mental status evaluation and the Hachinski Ischemic Scale are shown in Table 3. No differences were observed in is rates of dementia or cognitive decline as measured by serial assessment. The incidence of new stroke and TIA were similar between groups and there were no significant changes detected in the MMSE or HIS surveys. During this period of time, 4 patients underwent a cardioversion, 1 received a pacemaker, none underwent an ablation, and 6 were start on antiarrhythmic drug therapy.

**TABLE 3:** Primary and secondary endpoints at 24 months stratified by treatment arm

	Dabigatran, *n* = 32	Warfarin, *n* = 31	*p*‐value
*Primary*			
Dementia diagnosis	0 (0%)	0 (0%)	—
Cognitive impairment	5 (15.6%)	2 (6.5%)	.23
ADAS >30% increase	5 (15.6%)	2 (6.5%)	.23
DAD >30% decrease	0 (0%)	0 (0%)	—
DAD >50% incorrect	0 (0%)	0 (0%)	—
*Secondary*			
Stroke or TIA	2 (4.0%)	0 (0%)	.24
Stroke	0 (0%)	0 (0%)	—
TIA	2 (4.0%)	0 (0%)	.24
MMSE % change			
Mean ± SD	7.3 ± 46.9%	5.9 ± 27.4%	.89
Median	0%	0%	.56
HIS % change			
Mean ± SD	4.3 ± 8.9%	2.6 ± 8.3%	.43
Median	3.4%	0%	.50

Abbreviations: ADAS, Alzheimer's Disease Assessment Scale; DAD, Disability Assessment for Dementia; HIS: Hachinski Ischemic scale; MMSE, Mini‐Mental Status Evaluation.

**FIGURE 1:**
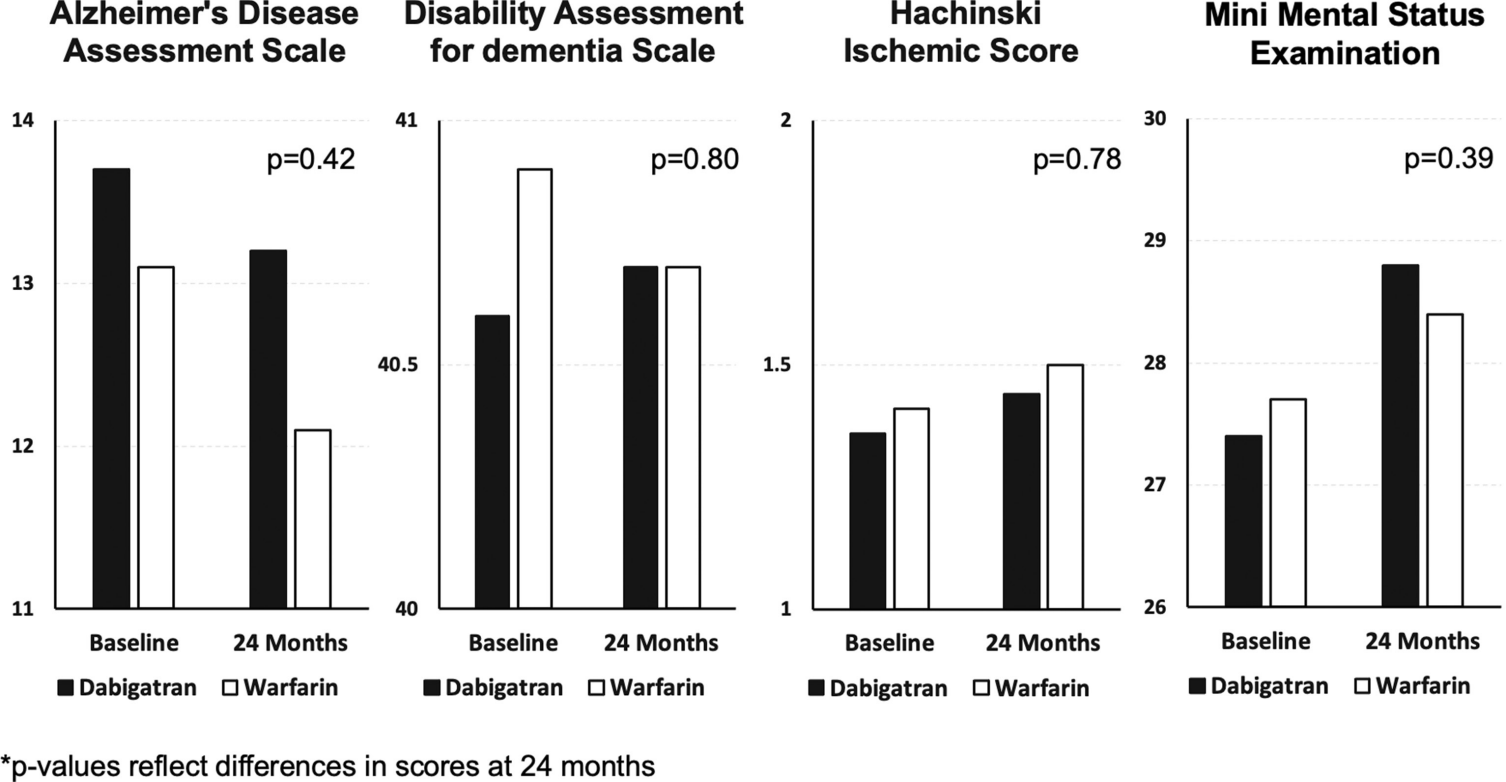
The survey scores of multiple tests for dementia, stroke, cognitive decline, and general function are shown and compared by initial treatment allocation to warfarin or dabigatran. There were no statistical differences observed at enrollment or study conclusion.

**FIGURE 2:**
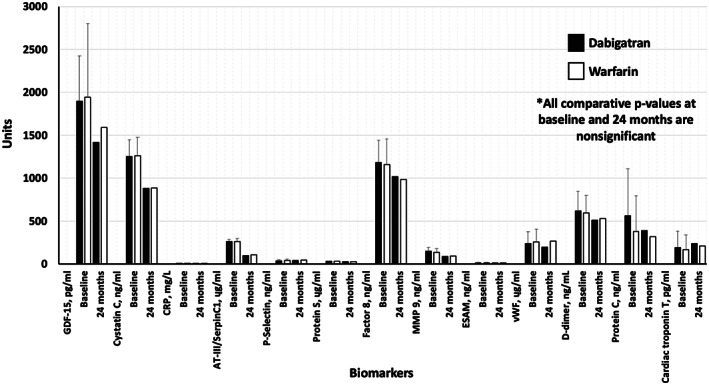
The figure shows a sample of biomarkers assessed at baseline and 24 months. There were no significant differences in values at baseline, 24 months, or in the magnitude of change between values between patients treated with dabigatran versus warfarin

The survey scores of multiple tests for dementia, stroke, cognitive decline, and general function are shown in Table 4 and shown for 4 surveys in Figure 1. The scores were similar at baseline and 24 months without significant differences observed in the dabigatran versus warfarin groups amongst all surveys in Figure 2.

**TABLE 4:** Baseline and 24‐month survey results stratified by treatment arm

	Dabigatran, *n* = 32	Warfarin, *n* = 31	*p*‐value
*ADAS*			
Baseline score among all participants, *n* = 91			
Mean ± SD	13.7 ± 5.8	13.1 ± 5.7	.61
Median	13.7	11.8	.44
Baseline score among participants completing study, *n* = 63			
Mean ± SD	13.2 ± 5.4	12.1 ± 5.0	.41
Median	13.3	11.3	.27
24‐month scores			
Mean ± SD	11.4 ± 7.0	10.0 ± 6.7	.42
Median	8.7	8.7	.60
Percent change from baseline to 24‐months			
Mean ± SD	−12.4 ± 38.3%	−21.2 ± 30.3%	.32
Median	−11.5%	−23.5%	.52
*DAD*			
Baseline score among all participants, *n* = 92			
Mean ± SD	40.6 ± 1.0	40.9 ± 0.4	.05
Median	41.0	41.0	.06
Baseline score among participants completing study, *n* = 63			
Mean ± SD	40.6 ± 1.1	40.9 ± 0.4	.18
Median	41.0	41.0	.24
24‐month scores			
Mean ± SD	40.7 ± 0.8	40.7 ± 0.9	.80
Median	41.0	41.0	.35
Percent change from baseline to 24‐months			
Mean ± SD	0.22 ± 3.33	−0.38 ± 2.47	.42
Median	0	0	.74
*HIS*			
Baseline score among all participants, n = 96			
Mean ± SD	1.36 ± 0.99	1.41 ± 0.96	.82
Median	1.00	1.00	.82
Baseline score among participants completing study, n = 63			
Mean ± SD	1.44 ± 0.84	1.42 ± 0.99	.94
Median	1.00	1.00	.75
24‐month scores			
Mean ± SD	1.44 ± 0.72	1.50 ± 1.04	.78
Median	1.00	1.00	.81
Percent change from baseline to 24‐months			
Mean ± SD	4.3 ± 8.9%	2.6 ± 8.3%	.43
Median	3.4%	0%	.56
*MMSE*			
Baseline score among all participants, n = 96			
Mean ± SD	27.4 ± 2.7	27.7 ± 2.2	.51
Median	28.0	28.0	.82
Baseline score among participants completing study, n = 63			
Mean ± SD	27.7 ± 2.2	27.8 ± 2.3	.88
Median	28.0	28.0	.81
24‐month scores			
Mean ± SD	28.8 ± 1.8	28.4 ± 1.8	.39
Median	29.0	29.0	.28
Percent change from baseline to 24‐months			
Mean ± SD	7.3 ± 46.9%	5.9 ± 27.4%	.89
Median	0%	0%	.50

Abbreviations: ADAS, Alzheimer's Disease Assessment Scale; DAD, Disability Assessment for Dementia; HIS, Hachinski Ischemic scale; MMSE, Mini‐Mental Status Evaluation.

Finally, the results of the biomarker data are listed in Table 5 which were similar at baseline and 24 months that suggest similar efficacy of anticoagulation and consistent impact of it with both anticoagulation strategies. In addition, the data showed similar levels of Neprilysin and the single‐nucleotide polymorphism rs6656401 and Complement Receptor‐1gene that may have adversely impacted cerebral amyloid angiopathy risk. Additional APOE profiling was similar amongst groups and did not associate with abnormal cognitive survey scores at 24 months.

**TABLE 5:** Biomarker results stratified by treatment arm. Results are presented as mean ± SD (median)

	Dabigatran	Warfarin	*p*‐value
GDF‐15, pg/mL			
Baseline	1897.3 ± 1071.6 (1566.5)	1942.8 ± 916.2 (1605.9)	.60
24 months	1416.4 ± 529.6 (1416.8)	1592.4 ± 858.5 (1295.1)	.77
Percent change	−18.2 ± 27.1 (−18.0)	−16.2 ± 28.1 (−21.5)	.95
Absolute change	−510.6 ± 903.3 (−352.8)	−373.9 ± 546.9 (−337.2)	.98
Cystatin C, ng/mL			
Baseline	1251.6 ± 413.3 (1113.6)	1262.0 ± 308.2 (1250.1)	.42
24 months	879.3 ± 197.1 (809.3)	886.2 ± 215.7 (870.8)	.82
Percent change	−26.8 ± 16.4 (−25.2)	−27.1 ± 22.9 (−34.5)	.51
Absolute change	−376.7 ± 339.2 (−282.3)	−386.8 ± 363.5 (−412.2)	.49
Prothrombin fragments 1 + 2, nmol/L			
Baseline	1.1 ± 1.6 (0.5)	1.2 ± 1.6 (0.5)	.52
24 months	1.2 ± 1.0 (0.9)	1.6 ± 1.8 (1.1)	.98
Percent change	146.4 ± 370.1 (59.5)	123.7 ± 248.7 (43.8)	.94
Absolute change	−0.1 ± 2.0 (0.2)	0.3 ± 1.6 (0.2)	.80
CRP, mg/L			
Baseline	6.0 ± 4.67 (4.9)	4.9 ± 3.3 (4.7)	.54
24 months	2.5 ± 2.3 (1.3)	1.8 ± 1.4 (1.1)	.49
Percent change	−34.6 ± 68.8 (−56.4)	−23.9 ± 129.2 (−63.3)	.52
Absolute change	−3.5 ± 3.6 (−2.8)	−3.1 ± 3.3 (−2.8)	.75
Antithrombin‐III/SerpinC1, ųg/mL			
Baseline	261.7 ± 108.2 (263.4)	262.8 ± 90.2 (278.7)	.43
24 months	95.0 ± 27.9 (85.2)	104.0 ± 32.7 (94.8)	.29
Percent change	−49.7 ± 57.1 (−68.5)	−50.1 ± 39.3 (−65.2)	.63
Absolute change	−167.7 ± 113.3 (−169.4)	−165.2 ± 97.8 (−184.9)	.76
P‐Selectin, ng/mL			
Baseline	36.3 ± 9.3 (35.7)	41.2 ± 15.7 (38.1)	.36
24 months	41.6 ± 15.6 (40.3)	43.2 ± 13.9 (42.2)	.53
Percent change	16.0 ± 36.9 (12.7)	9.9 ± 30.8 (8.8)	.81
Absolute change	5.0 ± 13.5 (4.6)	1.5 ± 15.2 (2.5)	.75
Protein S, ųg/mL			
Baseline	29.1 ± 4.4 (27.6)	28.3 ± 4.3 (27.2)	.52
24 months	25.6 ± 5.2 (23.8)	25.2 ± 3.85 (25.4)	.97
Percent change	−10.8 ± 20.2 (−14.7)	−10.2 ± 18.9 (−16.9)	.82
Absolute change	−3.6 ± 6.5 (4.0)	−3.0 ± 5.7 (−5.0)	.84
Factor 8, ng/mL			
Baseline	1180.7 ± 149.0 (1210.3)	1156.9 ± 236.0 (1224.1)	.86
24 months	1019.7 ± 263.7 (1101.4)	984.5 ± 298.7 (1058.5)	.53
Percent change	−12.0 ± 25.7 (−9.8)	−12.5 ± 38.7 (−15.1)	.32
Absolute change	−153.6 ± 279.1 (−124.4)	−168.2 ± 248.7 (−5.0)	.44
MMP 9, ng/mL			
Baseline	148.3 ± 246.9 (95.1)	132.4 ± 123.6 (104.6)	.63
24 months	87.5 ± 45.1 (78.4)	93.5 ± 44.9 (85.1)	.42
Percent change	32.7 ± 127.4 (−7.8)	−2.7 ± 54.1 (−11.3)	.59
Absolute change	−62.0 ± 246.8 (−6.1)	−40.0 ± 123.8 (−7.4)	.83
Neprilysin, ng/mL			
Baseline	164.9 ± 408.4 (20.5)	52.8 ± 53.7 (30.8)	.95
24 months	337.8 ± 543.8 (26.1)	322.9 ± 436.3 (52.7)	.73
Percent change	239.6 ± 413.0 (42.9)	331.0 ± 639.0 (55.1)	.69
Absolute change	167.8 ± 506.9 (0.9)	253.0 ± 395.4 (0.6)	.98
BNP, pg/mL			
Baseline, *n* = 20	10.6 ± 8.4 (6.3)	9.3 ± 5.0 (10.8)	.55
24 months, *n* = 25	8.2 ± 9.2 (5.7)	6.2 ± 1.5 (5.8)	.76
Percent change, *n* = 11	55.2 ± 204.4 (−35.2)	−9.5 ± 86.8 (−50.9)	.84
Absolute change	−1.6 ± 17.8 (−3.4)	−1.3 ± 9.4 (−1.0)	.87
CCL23, pg/mL			
Baseline	1332.9 ± 1217.0 (830.5)	1014.9 ± 923.2 (474.2)	.45
24 months	643.2 ± 542.8 (419.7)	462.0 ± 269.6 (386.6)	.40
Percent change	−8.8 ± 91.3 (−25.8)	−9.4 ± 82.7 (−30.7)	.88
Absolute change	−725.5 ± 1292.5 (−147.3)	−584.3 ± 959.5 (−139.6)	.67
ESAM, ng/mL			
Baseline	12.6 ± 10.4 (9.8)	12.3 ± 9.8 (9.2)	.52
24 months	12.4 ± 8.8 (9.6)	10.1 ± 8.2 (7.7)	.08
Percent change	14.0 ± 61.6 (0.26)	−1.5 ± 83.9 (−21.4)	.09
Absolute change	−0.3 ± 9.9 (0.02)	−2.5 ± 5.6 (−1.9)	.06
vWF, ųg/mL			
Baseline	236.5 ± 196.0 (183.4)	255.2 ± 199.2 (237.4)	.72
24 months	192.9 ± 137.8 (164.3)	267.0 ± 149.4 (296.8)	.04
Percent change	401.8 ± 1498.7 (−28.3)	428.3 ± 1128.0 (42.0)	.38
Absolute change	−44.9 ± 265.9 (−29.9)	20.3 ± 256.2 (85.6)	.30
D‐dimer, ng/mL			
Baseline	617.3 ± 307.9 (536.4)	594.4 ± 249.7 (567.3)	.89
24 months	508.3 ± 230.2 (484.9)	527.8 ± 204.8 (458.8)	.75
Percent change	14.6 ± 139.1 (−21.4)	18.9 ± 160.3 (−21.9)	.92
Absolute change	−109.5 ± 327.8 (−77.1)	−70.8 ± 304.1 (−118.3)	.93
Protein C, ng/mL			
Baseline	562.7 ± 926.5 (250.7)	377.8 ± 467.8 (157.5)	.50
24 months	387.0 ± 548.5 (153.7)	317.6 ± 415.7 (104.8)	.16
Percent change	135.0 ± 386.3 (−25.9)	111.2 ± 595.4 (−41.6)	.57
Absolute change	−186.5 ± 648.1 (−49.1)	−72.7 ± 318.8 (−64.2)	.77
Cardiac troponin T, pg/mL			
Baseline	191.7 ± 124.8 (185.5)	167.6 ± 61.4 (133.0_	.23
24 months	235.7 ± 190.6 (180.9)	210.5 ± 171.2 (165.2)	.65
Percent change	63.7 ± 183.6 (18.9)	67.7 ± 131.4 (15.1)	.41
Absolute change	27.2 ± 145.6 (21.3)	34.9 ± 97.1 (19.2)	.71
AntiBeta 2 Glycoprotein I IgG, U/mL			
Baseline	101.1 ± 232.2 (70.1)	103.2 ± 51.3 (95.1)	.08
24 months	113.2 ± 158.5 (80.2)	78.4 ± 51.4 (74.1)	.46
Percent change	20.0 ± 88.8 (5.6)	−6.2 ± 80.0 (−36.1)	.19
Absolute change	−1.0 ± 50.8 (6.2)	−29.4 ± 77.1 (−26.1)	.10
AntiBeta 2 Glycoprotein I IgM, U/mL			
Baseline	117.8 ± 104.3 (75.5)	231.9 ± 339.6 (127.6)	.02
24 months	153.7 ± 162.2 (102.9)	170.8 ± 150.8 (132.4)	.46
Percent change	20.5 ± 81.6 (−8.1)	−1.0 ± 76.0 (−10.2)	.27
Absolute change	20.0 ± 90.0 (−7.3)	−63.0 ± 281.3 (−18.1)	.32
Anti‐Cardiolipin, U/mL			
Baseline	3.2 ± 2.0 (3.0)	4.7 ± 5.1 (3.3)	.45
24 months	3.5 ± 3.6 (3.2)	4.3 ± 3.7 (3.0)	.60
Percent change	209.9 ± 1073.0 (13.1)	22.9 ± 116.5 (−1.4)	.40
Absolute change	0.4 ± 3.0 (0.2)	−0.5 ± 3.9 (−0.1)	.64

Abbreviations: BNP, brain natriuretic peptide; CCL23, chemokine ligand 23; CRP, c‐reactive protein; ESAM, endothelial cell adhesion molecule; GDF‐15, growth differentiation factor 15; MMP, matrix metalloproteinase; vWF, von Willebrand factor.

In the subgroup of patients that underwent MRI testing, there were no new strokes (clinical or subclinical) identified during the study follow‐up or quantifiable change in volume (Supplemental Table S2).

## 4 DISCUSSION

As few therapies to halt progression of cognitive decline and dementia have been ineffective, attention has been turned towards identification of moderate‐high risk patients and early use of potential preventative treatments.^10^ In the setting of patients with AF, rhythm control and early use of anticoagulation are potential means to reduce the risk of cognitive decline and dementia.^14^

Before considering the findings of this study of anticoagulation for patients with AF, it is important to recognize this was a vanguard study and the event rates were lower than expected. As described above, we used event rates of prior observational studies to calculate a power to determine a difference in the primary endpoint. However observational trials have limitations and as such we prespecified in the protocol that this would be a vanguard study that would enroll 120 patients to “determine trends of cognition in the prospective study design that include incidence of dementia and the percent of patients that develop moderate decline in each study group to compare amongst the rates observed in the general population, the feasibility of recruitment, study design and subject retention, budget adherence and likelihood of obtaining the desired outcomes for potential future trial design and planning”. The neutrality of the outcomes and the biomarkers that showed very similar efficacy of both strategies were considered in the decision to not move forward with a larger, fully powered study based upon these randomized prospective data and their event rates.

Nonetheless, the current trial resulted in several key and interesting observations:

1. Anticoagulation use in this prospective randomized trial, whether with warfarin or dabigatran was associated with very low dementia and stroke rates. These findings with contemporary management for other associated cardiovascular disease states suggest that a trial to detect any differences would need to be much larger and likely require an extended follow‐up period beyond 2 years.
2. Serial cognitive testing results after anticoagulation initiation in did not demonstrate cognitive decline and trended towards modest improvement. We hypothesized that the generalized cognitive scoring improvement may reflect recall bias from serial testing,^15^ improved follow‐up and management, and perhaps influence of the anticoagulant as it related to brain perfusion with a reduction in risk of ischemic brain injury over time.
3. Anticoagulation use in patients results in a durable reduction of thrombotic and ischemic biomarkers that have been shown, when elevated, to be associated with the risk of stroke and vascular injury. However, similar to many trials of anticoagulation and population‐based studies of anticoagulation use, long‐term use and compliance remains a challenge.^5^, ^6^, ^7^ In this study approximately 1/3 discontinued their anticoagulation for a variety of reasons. Underuse has clearly been shown to increase risk of stroke. In this analysis, those at discontinued therapy also impacted our ability to study both therapies as to how they relate to anticoagulation.^16^
4. Stroke, dementia, and cognitive outcomes were similar with both routine start prescription of dabigatran or CPAS‐directed warfarin anticoagulation in patients with AF at 2 years.

We observed a lower rate of moderate–severe cognitive decline and dementia than expected based on findings from a routine population of patients with AF treated with warfarin.^22^ These lower rates may be from early and effective use of anticoagulation after AF diagnosis that has been shown to lower dementia risk as this trial involved all patients referred for AF management that included a goal of anticoagulation initiation.^23^ It is also possible that the relatively small patient population, with approximately 1/3 discontinuing anticoagulation, rendered the trial unable to detect a true incidence within the defined study follow‐up period. Finally, in a prospective, randomized trial with frequent follow‐up visits the outcomes may be different due to more frequent assessment of drug efficacy, identification and management of risk factors for AF‐related comorbidities, more close management of anticoagulation, and selection bias that occurs in patients that are willing to enroll in these types of studies.

These data are complementary to those recently presented from the GIRAF (CoGnitive Impairment Related to Atrial Fibrillation) trial which was a two‐year randomized, multi‐center, prospective trial in Brazil that evaluated the effects of dabigatran and warfarin on cognitive and functional impairment, bleeding, and cerebrovascular complications in patients (>70 years of age) with atrial fibrillation.^24^ In this trial, patients underwent 90‐min cognitive and functional evaluations at the one‐year and two‐year follow‐up visits as well as MRI brain imaging. At the conclusion of the trial, there were no patients that developed dementia and cognitive scoring assessments were identical in both treatment groups (<1 point difference). The study investigators concluded, similar to our study, that either strategy was acceptable as means to treat patients with AF to lower stroke risk and that neither can be used to preferentially impact risk of cognitive decline. These data combined with ours now comprise over 250 patients with serial testing of cognition after anticoagulation initiation with either dabigatran or warfarin.

Our data provide some additional mechanistic understanding into the equivalent outcomes when the biomarkers are considered. In our analysis, multiple markers of vascular injury, thrombosis, and inflammation were similar throughout the study in both treatment groups. This would suggest that both anticoagulation strategies were highly effective at the primary goal of reducing risk of micro and macro thromboembolism with reasonable safety of use. These data also support very well managed patients on warfarin anticoagulation. Highly effective warfarin can be accomplished in centers such as ours that use a dedicated \community pharmacy anticoagulation service (CPAS) guidance and administration; however in the general community both general warfarin use and compliance to DOAC therapies can be less optimal and the result in higher rates of brain injury.^25^, ^26^ In this community setting of less efficacy, a drug with a larger therapeutic window compared to warfarin may improve outcomes that were explored in this analysis.

The study has several limitations. First, this was a vanguard analysis and as such was underpowered to detect a significant difference in the endpoints considered in this analysis. This study design was pursued to inform on a subsequent study design, however the lack of any signal of difference and nearly equivalent outcomes between anticoagulation strategies in this analysis did not affirm a decision to perform a larger study; as such there was value in this study design and the results. Next, common to all randomized prospective and observational anticoagulation studies, long‐term adherence was suboptimal, and expected, and reflects many decisions including a desire to not use anticoagulation therapy in general. In a recent community‐based trial of predictors of contemporary oral anticoagulants use and adherence, persistence use at 2 years was approximately 80% and continued to steadily decline to approximately 70% at 4 years. In this analysis, lack of adherence significantly correlated with higher rates of stroke and stroke‐related mortality.^16^ Finally, patients were enrolled that were willing and able to complete the battery of cognitive tests. This may have selected a patient population that would perform better on these tests compared to a general population and long‐term results as well can be influenced by sequential testing and learned responses.

## 5 CONCLUSIONS

In this prospective randomized vanguard trial of patients with AF at moderate‐high risk of stroke, both the use of dabigatran and warfarin were associated with similar risks of stroke, cognitive decline, and dementia at 2 years suggestive that either strategy is acceptable to mitigate these risks. These outcomes must be considered in the setting of a relatively small study population and low incidences of stroke, dementia, and cognitive decline that can result in a type 2 error of data interpretation. However, these data combined with those from the GIRAF study suggest, at this time, that there is no preferential benefit to dabigatran compared to well‐managed warfarin therapy in patients with AF receiving a new start of anticoagulation to lower risk of cognitive decline and dementia at 2 years. As a Vanguard study the data did not support the pursuit of a subsequent larger, adequately powered study.

However, as mentioned previously, warfarin management in the community is clearly different than in randomized control trials and when used in routine clinical practice a preferential benefit with DOAC therapy use may become more apparent. In addition, long‐term data beyond the scope of a a randomized clinical trial can be obtained. At this point there is a need to return to this level of evidence to construct subsequent trials. In addition, as dabigatran has a discrete anticoagulation mechanism compared to other DOAC therapies, also explore alternative anticoagulants in an effort to improve brain health if patients with AF.

## FUNDING INFORMATION

Funding provided by Boehringer Ingelheim.

## CONFLICT OF INTEREST

TJB: Research grants: Altathera, Boehinger Ingelheim, Boston Scientific, Heartline Study Steering Committee for Janssen Scientific Affairs/Johnson & Johnson, HM: None, MJC: Heartline Study Steering Committee for Janssen Scientific Affairs/Johnson & Johnson, SCW: None, VJ: None, SMS: None, JC: None, KUK: none, JBM: None, BAS: National Heart, Lung, And Blood Institute of the National Institutes of Health (#K23HL143156), and reports research support from Abbott, Boston Scientific, and Janssen; and consulting to Janssen, AltaThera, Merit Medical, and Bayer; and speaking for NACCME (funded by Sanofi), JLA: None.

## ETHICS APPROVAL STATEMENT

The trial was a single center trial that will be conducted at Intermountain Medical Center in Murray, Utah. The Intermountain Medical Center Cardiovascular Executive Committee and Internal Review Board approved the study. A data safety monitoring board was created, and reviewed patient events and study progress every 6 months or sooner on an as needed basis.

## PATIENT CONSENT STATEMENT

All patients consented to participate in the trial under the direction of the Intermountain Medical Center Cardiovascular Executive Committee and Internal Review Board. Participation was voluntary and patients could withdraw from participation at any time without penality or impact on their clinical care.

## CLINICAL TRIAL REGISTRATION

The Cognitive Decline and Dementia in Atrial Fibrillation Patients (CAF) Trial (ClinicalTrials.gov Identifier: NCT03061006).

## Supporting information


Table S1
