# Supplementary material for: Impact of anticoagulation therapy on the cognitive decline and dementia in patients with non‐valvular atrial fibrillation (cognitive decline and dementia in patients with non‐valvular atrial fibrillation [CAF] trial)
Source: J Arrhythm. 2022 Sep 19;38(6):997–1008. doi: 10.1002/joa3.12781 (PMC9745454; doi:10.1002/joa3.12781)
Supplement: Supplementary file 1 — Table S1 [file JOA3-38-997-s001.docx]

**Supplemental Files**

**Table 1**. Total number of patients who completed study (dabigatran vs. warfarin, p=0.74) and reason why patients didn’t complete the study stratified by treatment arm.

|  | **Dabigatran** | **Warfarin** |
| --- | --- | --- |
| Number completed study | 32 (64.0%) | 31 (60.8%) |
| *Reasons didn’t complete study* | | |
| Didn’t want to participate in study visits | 4 | 2 |
| Didn’t want to take an oral anticoagulant anymore | 5 | 10 |
| Wanted to switch to a direct oral anticoagulant | 0 | 2 |
| Worsening renal function | 1 | 0 |
| Lost to follow-up | 1 | 1 |
| Died | 3 | 1 |
| AE | 3 | 1 |
| Unknown | 0 | 3 |
| Lack of health insurance | 1 | 0 |

**Table 1 Legend**. The table shows the reasons for discontinuation of anticoagulation during stury enrollment. Of the patients enrolled in the study, 64% of those randomized to dabigatran and 61% of those randomized to warfarin completed the study.

**Table 2**. MRI results at baseline and 24 months stratified by treatment arm.

|  | **Dabigatran** | **Warfarin** | **p-value** |
| --- | --- | --- | --- |
| Baseline MRI |  |  |  |
| New lesion | 9.1% (1/11) | 0% (0/12) | 0.48 |
| Old lesion | 9.1% (1/11) | 9.1% (1/11) | 1.00 |
| 24 months |  |  |  |
| New lesion | 0% (0/6) | 0% (0/5) | --- |
| Old lesion | 0% (0/6) | 0% (0/5) | --- |

**Table 2 Legend**. The table shows the results of brain imaging by MRI at study enrollment and at 24 months for study conclusion. In this subgroup of study patients that underwent MRI testing, there were no new strokes (clinical or subclinical) identified during the study follow-up or a quantifiable change in volume
